# Phenotypic Variability in a Family with Acrodysostosis Type 2 Caused by a Novel PDE4D Mutation Affecting the Serine Target of Protein Kinase-A Phosphorylation

**DOI:** 10.4274/jcrpe.4488

**Published:** 2017-12-15

**Authors:** Julia Hoppmann, Julia Gesing, Caroline Silve, Chrystel Leroy, Astrid Bertsche, Franz Wolfgang Hirsch, Wieland Kiess, Roland Pfäffle, Volker Schuster

**Affiliations:** 1 University of Leipzig, Hospital for Children and Adolescents, Department of Women and Child Health, Leipzig, Germany; 2 Université Paris-Sud Faculté de Médecine, INSERM U1169, Département de Génétique et de Biologie Moléculaire, Le Kremlin Bicêtre, France; 3 Centre de Référence des Maladies Rares du Métabolisme phosphocalcique, Filiere Maladies Rares OSCAR, Assistance Publique Hôpitaux de Paris, Hôpital Cochin, Service de Génétique et Biologie Moléculaires, Paris, France

**Keywords:** Acrodysostosis, skeletal dysplasia, brachydactyly, inactivating parathyroid hormone/parathyroid hormone related protein signalling disorder, phosphodiesterase 4D

## Abstract

Acrodysostosis is a very rare congenital multisystem condition characterized by skeletal dysplasia with severe brachydactyly, midfacial hypoplasia, and short stature, varying degrees of intellectual disability, and possible resistance to multiple G protein-coupled receptor signalling hormones. Two distinct subtypes are differentiated: acrodysostosis type 1 resulting from defects in protein kinase type 1-α regulatory subunit and acrodysostosis type 2 caused by mutations in phosphodiesterase 4D (PDE4D). Most cases are sporadic. We report on a rare multigenerational familial case of acrodysostosis type 2 due to a novel autosomal dominantly inherited PDE4D mutation. A 3.5-year-old boy presented with short stature, midfacial hypoplasia, severe brachydactyly, developmental delay, and behavioural problems. Laboratory investigations revealed mild thyrotropin resistance. His mother shared some characteristic features, such as midfacial hypoplasia and severe brachydactyly, but did not show short stature, intellectual disability or hormonal resistance. Genetic analysis identified the identical, novel heterozygous missense mutation of the PDE4D gene c.569C>T (p.Ser190Phe) in both patients. This case illustrates the significant phenotypic variability of acrodysostosis even within one family with identical mutations. Hence, a specific clinical diagnosis of acrodysostosis remains challenging because of great interindividual variability and a substantial overlap of the two subtypes as well as with other related Gsα-cAMP-signalling-linked disorders.

**What is already known on this topic?**

Acrodysostosis is a rare congenital multisystem condition characterized by skeletal dysplasia, varying degrees of intellectual disability, and possible resistance to multiple G protein-coupled receptor signalling hormones. Acrodysostosis type 2 is caused by mutations in cAMP-specific phosphodiesterase 4D (PDE4D). To date, 26 different PDE4D gene mutations have been reported in humans.

**What this study adds?**

Identification of a novel heterozygous missense mutation of the PDE4D gene c.569C>T (p.Ser190Phe) in a familial case of acrodysostosis type 2 affecting the serine target of protein kinase-A phosphorylation within the motif RRESF.

Evidence for a significant phenotypic variability of acrodysostosis in patients carrying the same mutation.

## INTRODUCTION

Acrodysostosis is a very rare congenital multisystem condition characterized by (1) skeletal dysplasia with severe brachydactyly, midfacial hypoplasia, and short stature, (2) varying degrees of intellectual disability, and (3) possible resistance to multiple G protein-coupled receptor (GPCR) signalling hormones, including parathyroid hormone (PTH) and thyrotropin (TSH) (1,2). The syndrome was first described by Robinow et al (3) in 1971. Recently, genetic defects in cAMP-dependent protein kinase type 1-α regulatory subunit (PRKAR1A) and cAMP-specific phosphodiesterase 4D (PDE4D) were identified in patients with acrodysostosis by candidate gene analysis (4) and exome sequencing (2,5) respectively.

Since then, two distinct genetic and phenotypic subtypes of acrodysostosis have been differentiated: acrodysostosis type 1 resulting from defects in PRKAR1A, and acrodysostosis type 2 caused by mutations in PDE4D. PRKAR1A as well as PDE4D play a crucial role in the GPCR-Gsα-cAMP-protein kinase A-signalling pathway (cAMP/PKA-pathway), which is present in almost all cell types and mediates a wide spectrum of biological functions, i.e. the physiological effects of several hormones (6).

In acrodysostosis type 1, the mutations in the regulatory subunit of PRKAR1A impair signalling through GPCR that use Gsα/adenyl cyclase/cAMP/PKA as a major signalling pathway, including PTH related protein (PTHrP), known to play an important role in endochondral bone development (7). In the case of acrodysostosis type 2, the presence of skeletal abnormalities whose characteristics and severity are indistinguishable from those seen in acrodysostosis type 1 supports the idea that during development, signalling by PTHrP through the PTHR-cAMP-PKA pathway is also attenuated and suggests that the mutations in PDE4D observed in this disease ultimately result in inappropriately increased PDE activity. The finding that injection into zebrafish of human PDE4D carrying mutations causing acrodysostosis type 2, but not wild-type PDE4D, produces developmental abnormalities is consistent with this idea (1). Many splice variants of PDE4D require PKA-induced phosphorylation to achieve maximal enzymatic activity (8,9,10). Based on the location of PDE4D mutations occurring in acrodysostosis type 2, it has been suggested that they may influence regulation by PKA (11). In contrast, mutations that would inactivate PDE4D (e.g. nonsense mutations, deletions, or mutations disrupting the catalytic site) cause a different phenotype, which is, in certain respects, a mirror-image of abnormalities seen in acrodysostosis type 2 (1). Another group, however, reported that PDE4D mutations causing acrodysostosis type 2 resulted in impaired enzyme activity, and that the observed phenotype resulted from an over-compensatory increased expression of other PDE4 isoforms (12,13). Thus, although the precise nature of the effect of PDE4 mutations in this disease remains controversial, all currently available evidence favours the conclusion that global PDE activity is inappropriately increased.

The vast majority of the cases of acrodysostosis occur sporadically, most probably due to de novo point mutations with evidence of a paternal age effect (5,14,15). Only very few families with an autosomal dominant inheritance of acrodysostosis type 2 have been reported so far (15,16).

Here, we report on a novel autosomal dominantly inherited heterozygous PDE4D mutation in a multigenerational family with acrodysostosis that illustrates the clinical variability of this syndrome even within one family. In doing so, we aim to raise awareness for this rare and clinically heterogeneous disease.

## CASE REPORTS

### Patient 1

Patient 1 was born at 38 weeks’ gestation by caesarean section because of oligohydramnion and pathological cardiotocography. At birth, he was small for gestational age with a weight of 2530 g [4.5th percentile, standard deviation score (SDS) -1.68] and a length of 48 cm (7th percentile, -1.47 SDS). During the neonatal period, the patient had feeding problems and several episodes of hypoglycemia occurred. Initial psychomotor milestones were reached rather late, but still within the normal range (walking at 1.6 years of age, speaking single words at 1.5 years of age). However, subsequently, he displayed a delay in motor development and regression of verbal development, and ergotherapy and speech therapy were started. Adenoidectomy and insertion of tympanic ventilation tubes were performed at the age of 3 years.

**Figure 1:**
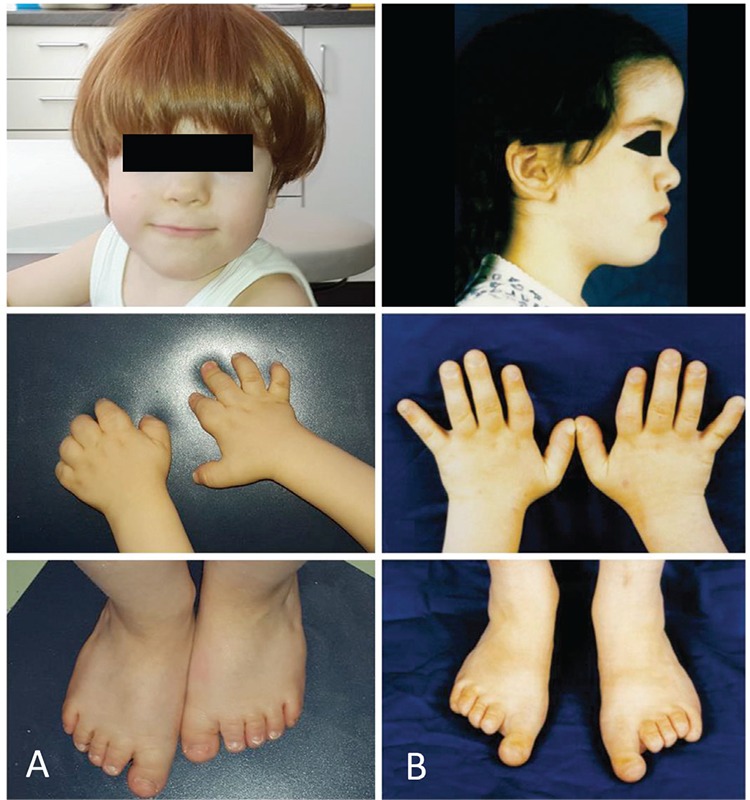
Photographs of the face, hands, and feet of patient 1 at the age of 3 years (A) and patient 2 at the age of 8 years (B). Note the facial dysostosis with flattening of the nasal bridge and the small broad hands and feet with relative sparing of the first toe [Figure 1B reproduced with permission of Springer (17)]

**Figure 2:**
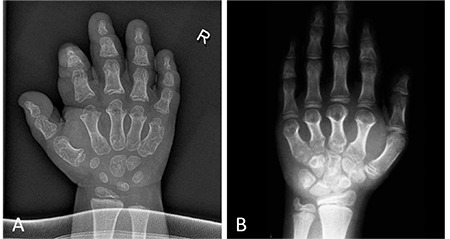
X-rays of the hands of patient 1 (A) and patient 2 (B). Note the severe brachydactyly with shortening of metacarpals and phalanges and cone-shaped epiphyses [Figure 2B reproduced with permission of Springer (17)]

At the age of 3.5 years, he was referred to our clinic for further clinical evaluation. On examination, he had light-coloured blue eyes and red-coloured hair, a round face with widely spaced eyes and midfacial hypoplasia with flattening of the nasal bridge (Figure 1A). Moreover, he had small broad hands and feet with stubby digits with relative sparing of the first toe. Furthermore, pigmented skin lesions were noticed on the upper arms and legs. Neuropsychological examination showed behavioural problems and a delay of gross and fine motor skills and speech development with severely impaired speech comprehension and dyslalia. He had grown below the 3^rd^ percentile since the age of 2 years with a height of 90.7 cm (0.9^th^ percentile, SDS -2.38) at the time of presentation. His weight was 12.8 kg (5.4^th^ percentile, SDS -1.61) with a body mass index of 15.6 kg/m^2^ (51.6th percentile, SDS +0.04). Radiographic examination showed severe brachydactyly with shortening of metacarpals, metatarsals and phalanges, except for the big toe, cone-shaped epiphyses, and a significantly advanced bone age (Figure 2A). Laboratory investigations showed normal results, except for a mildly elevated TSH of 7.98 mU/L, while thyroid hormone levels were normal.

### Patient 2

Patient 2 is the mother of patient 1 (17). She presented at the age of 8.8 years. At presentation, she had macrocephaly with frontal bossing, midfacial hypoplasia, and small broad hands and feet with brittle nails (Figure 1B). The big toe of both feet appeared hyperplastic. The patient had a height between the 3^rd^ and 10^th^ percentiles, a body weight at the 50^th^ percentile, and a head circumference above the 97^th^ percentile. Radiographs showed severe brachydactyly with short metacarpals II-V, metatarsals II-IV and phalanges, first ray hyperplasia of the foot, cone-shaped epiphyses, and early epiphyseal fusion (Figure 2B). Bone age was accelerated by 3 years. Magnetic resonance imaging of the head showed a thickened calvarium, as well as a 2 cm supracerebellar arachnoid cyst and a lipoma at the corpus callosum as incidental findings. Furthermore, a heart defect with septal aneurysm and atrioventricular valve defect was diagnosed. At initial presentation, laboratory investigations showed normal thyroid function tests, a normal PTH, and adequate calcium and phosphate levels. In the further course, she developed autoimmune thyroiditis as well as vitamin D deficiency. According to the medical history, she had started speaking at the age of 4 years. However, her further mental development was normal. She graduated from high school and momentarily studies law at the university.

Clinical, radiological and laboratory characteristics of patient 1 and 2 are summarized in Table 1.

Similar dysmorphic clinical findings and radiological features of the hands and feet were reported in the mother of patient 2 (grandmother of patient 1). She had died from leukemia. Other family members have not been affected.

Molecular analysis in patient 1 and patient 2 identified a novel heterozygous missense mutation of the PDE4D gene (NM-001104631): c.569C>T (p.Ser190Phe) in exon 2 and thereby confirmed the clinical diagnosis of acrodysostosis type 2. Interestingly, this mutation affects the serine target of PKA phosphorylation within the motif RRESF.

Informed consent was obtained from the patient’s family.

## DISCUSSION

In this article, we reported a very rare case of autosomal dominantly inherited acrodysostosis type 2 in a three-generational family caused by a novel mutation in the PDE4D gene illustrating the significant phenotypic variability of acrodysostosis and discuss differential diagnosis. Here by, we aim to increase the general awareness of this rare condition.

To our knowledge, this is the first report on a family with three generations to be affected by acrodysostosis type 2 due to a heterozygous missense mutation of the PDE4D gene c.569C>T (p.Ser190Phe) affecting the upstream conserved region 1 of the PDE4D. It is the second mutation affecting the serine target of PKA phosphorylation within the motif RRESF. To date, 26 missense mutations in PDE4D gene associated with acrodysostosis type 2 have been identified (including the novel mutation in the family discussed in this case report). All mutations described so far were sporadic, heterozygous de novo mutations, except for very few families with a genetically proven autosomal dominant inheritance of PDE4D mutations (15,16).

Second, in the present case, the phenotypic variability of affected family members sharing the identical mutation is striking. Interestingly, patient 1 was born small for gestational age and has short stature, whereas his mother (patient 2) who carried the same mutation has a normal height. Light-coloured blue eyes and red-coloured hair, originally described by Niikawa et al (18) in patients with acrodysostosis, were striking on clinical examination of patient 1 (15). In contrast, his mother presented with brown hair colour. Furthermore, patient 1 displayed behavioural problems and a delay of motor and speech development with severely impaired speech comprehension and dyslalia, whereas his mother graduated from high school and attends university. Patient 1 had mild TSH resistance, whereas his mother had normal thyroid functions tests at presentation. Clinical heterogeneity for acrodysostosis was also described by Lynch et al (15) in a family with three affected siblings whose father was found to have only subtle features of acrodysostosis but also carried the same mutation. Lynch et al (15) also found a high variability of intellectual abilities in this family. As possible explanations for the lack of full clinical expression, variable expressivity, mosaicism, and tissue specific imprinting of the gene are discussed (15).

Third, given the very low number of familial cases reported so far and the significant phenotypic variability of affected family members, it is most likely that some affected individuals are not considered for diagnosis because of subtle phenotypic features. Hence, familial cases might be underestimated. Therefore, careful clinical evaluation of the parents of affected patients for features of acrodysostosis is crucial to detect possible mutation carriers, to reveal new familial cases, and to gain information about the genotype-phenotype correlation in the future.

Fourth, considering the great clinical overlap between the subtypes of acrodysostosis and also with other Gsα-cAMP-signalling-linked disorders, a specific clinical diagnosis still remains difficult and challenging, if not even impossible.

In the past, the two subtypes of acrodysostosis were mainly differentiated by the presence or absence of hormonal resistance that was exclusively attributed to PRKAR1A mutations (19). However, Lindstrand et al (1) recently found that PDE4D mutations as well may lead to clinically significant endocrine abnormalities like PTH or TSH resistance in a small subset of patients (16). Moreover, the present case shows that even family members with the identical mutation in PDE4D might differ in the presence of hormonal resistance, illustrating the phenotypic variability. Therefore, endocrine follow-up remains important for all patients, regardless of the subtype of acrodysostosis. However, Elli et al (16) described different frequencies of phenotypic characteristics according to the mutated gene. Short stature and cone-shaped epiphyses were more often described in acrodysostosis type 1, whereas a more characteristic facial dysostosis, mental or behavioural defects, cryptorchidism and/or lack of pubertal spurt were found more often in acrodysostosis type 2 (16). Several non-mutually exclusive explanations for the phenotypic differences have been advanced, but further work is required to validate their importance (13,16,19,20). However, since the number of published cases of acrodysostosis, in particular type 2, is still low and single cases had a high impact on this comparative analysis, future alterations of observed clinical features are likely. A single distinctive feature that allows clear clinical differentiation of both subtypes has not been revealed yet.

Differential diagnosis should not only include the different subtypes of acrodysostosis but also other entities of the “inactivating PTH/PTHrP signalling disorders” that clinically present with Albright hereditary osteodystrophy (AHO) (21,22). AHO was first described by Albright et al (23) in 1942 and comprises heterogeneous clinical features such as brachydactyly, rounded face, short stature, stocky build and subcutaneous ossifications. In pseudohypoparathyroidism type 1A and type 1C, AHO is associated with hormonal resistance as well as obesity and varying degrees of intellectual disability. They are caused by maternal loss-of-function mutations or imprinting defects in the GNAS gene encoding Gsα. AHO without any evidence of hormonal resistance, called pseudopseudohypoparathyroidism, is due to paternal loss-of-function mutations in GNAS. Because of substantial clinical overlap of acrodysostosis with other related Gsα-cAMP-signalling-linked disorders, a clear clinical diagnosis without genetic analysis remains difficult.

In summary, we identified a novel heterozygous PDE4D mutation in a family with acrodysostosis type 2. This case illustrates the phenotypic variability of acrodysostosis even within one family with identical mutations. We conclude that a specific clinical diagnosis of acrodysostosis remains challenging because of great interindividual variability and a substantial overlap of the two subtypes as well as with other related Gsα-cAMP-signalling-linked disorders.

## Figures and Tables

**Table 1:**
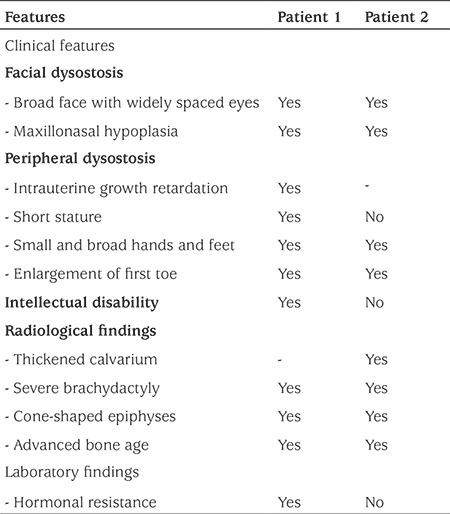
Clinical, radiological, and biochemical features in the patients with acrodysostosis
